# ACMNet: An attention-convolution mixed neural network for semantic segmentation of high-resolution remote sensing images

**DOI:** 10.1371/journal.pone.0358021

**Published:** 2026-11-06

**Authors:** Shangkun Yu, Leijuan Yu, Tingting Wang, Rui Hu, Chengxin Wang

**Affiliations:** 1 School of Geography and Tourism, Qilu Normal University, Jinan, China; 2 College of Geography and Environment, Shandong Normal University, Jinan, China; 3 School of Bioengineering, Shandong Polytechnic, Jinan, China; 4 Inspur Group Co., Ltd, Jinan, China; 5 Business School, University of Chinese Academy of Social Sciences, Beijing, China; 6 Key Research Institute of Yellow River Civilization and Sustainable Development & Yellow River Civilization by Provincial and Ministerial Co-construction of Collaborative Innovation Center, Henan University, Kaifeng, China; PLOS, UNITED KINGDOM OF GREAT BRITAIN AND NORTHERN IRELAND

## Abstract

Semantic segmentation of remote sensing imagery, which is widely used in land cover classification, urban planning, and environmental monitoring, has become a research hotspot in the fields of remote sensing and computer vision. As its applications continue to expand, improving the segmentation accuracy and generalization have become pressing challenges. However, most existing semantic segmentation models rely solely on either attention mechanisms or convolutional neural networks (CNNs), thus leaving room for performance improvement. In this study, we propose ACMNet (Attention-Convolution Mixed Network), which adopts a hybrid attention-convolution architecture. The decoder of ACMNet employs an innovative mixed design, where two branches are used to extract and integrate different types of contextual information and achieve hierarchical aggregation of contextual features, thereby enhancing its performance. To evaluate the performance of ACMNet, we conducted ablation experiments and comparative experiments on the Potsdam, Vaihingen, and WHU Building datasets. The results show that ACMNet achieved the best overall performance across all three datasets, with mF1 scores of 91.79%, 88.70%, and 96.56% on the Potsdam, Vaihingen, and WHU Building datasets, respectively. These findings indicate that ACMNet delivers superior performance in the semantic segmentation of high-resolution remote sensing images in urban areas, which provides valuable support for diverse tasks related to fine-grained urban management and dynamic monitoring.

## Introduction

With the rapid advancement of remote sensing technology, particularly the launch of high-resolution, multispectral, and hyperspectral satellites, the spatial resolution of re-mote sensing imagery has been significantly improved [1,2]. The wide application of such high-resolution imagery has become an indispensable part of modern scientific research and practical applications. Such imagery contains abundant information about ground objects and is highly diverse and complex, which enables fine-grained land cover classification and change monitoring [3]. However, due to large intra-class variance, small inter-class variance, and low inter-class separability, traditional image processing methods struggle to accurately identify and segment different ground objects [4]. Se-mantic segmentation aims to divide pixels or regions in remote sensing imagery into classes with specific semantic labels, such as roads, buildings, water bodies, and vegetation [5,6]. This technique has a broad range of applications, including land cover classification [7,8], urban planning [9,10], environmental monitoring [11], and disaster assessment [12,13]. The rapid development of computer vision, particularly the emergence of deep learning, has provided powerful technical support for the semantic segmentation of remote sensing imagery [14]. As such, semantic segmentation has attracted extensive attention from researchers in the interdisciplinary fields of computer vision and remote sensing [5,6] and has become a key approach to overcoming the limitations of traditional image processing techniques.

Currently, convolutional neural network (CNN)-based semantic segmentation methods generally adopt an encoder-decoder architecture as their backbone [15,16]. Although this structure has achieved promising results in semantic segmentation tasks, several challenges remain. These include limited capability to model fine-grained details and contextual information, as well as low segmentation accuracy along object boundaries. Since convolutional kernels mainly extract features within local neighborhoods, CNNs are better suited to capturing textures, edges, and local shape information, but they remain limited in modeling semantic relationships between distant regions. In high-resolution remote sensing images, objects of the same class may be distributed across a large spatial extent, while objects from different classes may show similar spectral or textural characteristics. Relying solely on local convolution can therefore lead to problems such as fragmented building boundaries, confusion between low vegetation and trees, and missed detection of small objects. Therefore, introducing an attention mechanism capable of modeling long-range dependencies can supplement global contextual information from a holistic perspective, which helps improve the discriminative power and semantic consistency of feature representations [17,18].

In semantic segmentation tasks, the incorporation of attention mechanisms enhances a model’s ability to capture key regions and salient features. By dynamically adjusting the weights of different spatial locations or channels in the feature map, attention mechanisms guide the model to focus on regions with higher semantic relevance to the task. De-pending on the dimension of operation, attention mechanisms can be categorized into spatial attention, channel attention, and self-attention. Spatial attention emphasizes “where is important” and assists the model in locating salient regions; channel attention focuses on “which features are important” and reinforces critical semantic channels; and self-attention models the dependencies between any two positions in the image, thereby enhancing the capability of global context modeling. By integrating attention mechanisms into the encoder, decoder, or multi-scale feature fusion modules, semantic seg-mentation models can more precisely delineate object boundaries, improve the recognition of small objects and complex backgrounds, and significantly boost overall seg-mentation performance [19]. Nevertheless, existing attention-based models still face challenges such as high computational cost, insufficient recognition of small objects, and limited capability in cross-scale information modeling, which constrain their generalization in complex scenarios.

While CNNs demonstrate strong performance in modeling local features, attention mechanisms exhibit clear advantages in modeling long-range dependencies. Specifically, the local receptive fields of CNNs make it difficult for them to directly capture dependencies between distant pixels or regions. This limitation is especially evident when processing large-scale buildings, continuous roads, and areas where tree canopies and low vegetation are intermingled, as the lack of global semantic context can easily lead to class confusion. By calculating correlations between different spatial positions or channels, self-attention mechanisms can introduce broader contextual information for each position, thereby alleviating the limitations of CNNs in long-range dependency modeling and semantic consistency preservation. However, relying solely on attention mechanisms may weaken the representation of local textures, boundary contours, and small-object details, while also introducing substantial computational overhead. Therefore, a key challenge in current research is how to effectively integrate the complementary strengths of convolution and attention mechanisms to construct models that are both sensitive to local details and capable of global semantic understanding.

To address the challenges discussed above, in this study, we propose a hybrid architecture that integrates attention mechanisms with convolutional neural networks, namely, ACMNet (Attention-Convolution Mixed Network). Within the decoder, ACMNet incorporates a collaborative structure consisting of a self-attention branch and a convolution branch, which gives full play to the complementary strengths of global and local features. This design enhances the model’s capacity to understand complex scenarios and improves its segmentation accuracy. We first conduct ablation experiments on the ISPRS Potsdam dataset to assess the effectiveness of the model’s internal components. We then conduct comparative experiments on the Potsdam, Vaihingen, and WHU Building datasets to verify the applicability of the proposed method across datasets with different spatial resolutions and category compositions, as well as in building extraction tasks.

## Related work

### CNNs

CNNs are a deep learning architecture that have demonstrated remarkable performance across a variety of domains, including image processing, video analysis, and natural language processing. A CNN is primarily composed of three types of layers: convolutional layers, pooling layers, and fully connected layers. Each type of layer performs a different function, and together they enable the transformation from raw image data to the final classification output.

The convolutional layer is the core component of a CNN, primarily responsible for feature extraction. In this layer, small convolution kernels (or filters) slide over the input image and compute the dot product between the kernel and local regions of the image to generate a feature map. By employing multiple convolution kernels, the network can extract diverse features within the same layer. Typically, a nonlinear activation function, such as the rectified linear unit (ReLU), is applied after each convolutional layer. The activation function introduces nonlinearity into the network and enables CNNs to learn more complex features. Following the convolutional layers, pooling layers are usually applied to reduce the spatial dimensions of feature maps, thereby decreasing the number of parameters and computational cost. The most common pooling operations are max pooling and average pooling, which output the maximum and average values within a region, respectively. After multiple convolutional and pooling layers, CNNs generally include one or more fully connected layers. These layers transform the outputs of the previous layers, typically a set of high-level features, into the final predictions. In fully connected layers, each input node is connected to every output node, resulting in a relatively large number of parameters. CNNs are trained using backpropagation and gradient descent, in which the weights of convolution kernels and fully connected layers are iteratively updated to minimize a loss function, such as cross-entropy loss. To improve training efficiency and mitigate overfitting, commonly employed techniques include weight initialization strategies, batch normalization, and dropout.

The most classical CNN-based model for semantic segmentation is the fully convolutional network (FCN) [20], which was the first to replace the fully connected layers of traditional CNNs with convolutional layers. This modification enabled the model to handle input images of arbitrary size and produce pixel-level predictions of corresponding dimensions. FCN also introduced skip connections that fuse shallow and deep features, thereby improving segmentation accuracy along object boundaries. Since then, numerous models have been developed based on FCN. For example, SegNet employs an encoder-decoder structure to enhance segmentation performance [21], while U-Net achieves accurate boundary segmentation through the use of skip connections [22]. More recently, increasing attention has been given to multi-scale information fusion to further improve the performance of semantic segmentation for remote sensing imagery. DeepLabV3 + , for instance, employs atrous convolution and pyramid pooling modules to capture multi-scale contextual information [23]. Building upon FCN, DeepLabV3+ in-corporates several innovations, including atrous convolution, atrous spatial pyramid pooling (ASPP), and multi-scale feature fusion. In addition, it employs residual connections and batch normalization, which together enable the model to achieve outstanding performance in semantic segmentation tasks.

### Attention mechanisms

Attention mechanisms are an important technique in deep learning, particularly in natural language processing and computer vision. Their core idea is inspired by humans’ visual attention, which selectively focuses on the most critical parts of information when processing large volumes of data. When applied to semantic segmentation of remote sensing imagery, attention mechanisms assign different weights to different regions of the image, whereby features that are more relevant to the segmentation task are emphasized while irrelevant or noisy information is suppressed, thereby improving the performance [24,25]. Typical categories of attention mechanisms include spatial attention, channel attention, and self-attention.

Spatial attention mechanisms transform various types of deformed data in the spatial domain and automatically capture important regional features. They ensure that images subjected to cropping, translation, or rotation can still yield results consistent with those of the original image [26,27]. The earliest spatial attention model was the Spatial Trans-former Network (STNet) [28], a neural network module capable of adaptively performing spatial transformations on input data. Given that remote sensing imagery often exhibits large scale, high dimensionality, and complex spatial structures, traditional image segmentation methods struggle to process such data effectively. STNet addresses this challenge by adaptively transforming input images in the spatial domain, thereby im-proving the accuracy and robustness of semantic segmentation. However, despite its advantages, STNet also has certain limitations. It requires large volumes of training data to learn transformation parameters; otherwise, the model may suffer from overfitting. In addition, its performance is less effective in handling complex transformations, such as nonlinear deformations.

In addition to spatial attention, channel attention mechanisms have also been widely applied in the semantic segmentation of remote sensing imagery. Channel attention focuses on the relative importance of feature channels by assigning different weights to each channel, thereby highlighting the most informative ones. This is typically achieved using global average pooling followed by fully connected layers [29,30]. The earliest channel attention model was the Squeeze-and-Excitation Network (SENet) [31], which consists of two main components: squeeze and excitation. The squeeze operation com-presses global spatial information and performs feature learning along the channel dimension, while the excitation operation adaptively assigns weights to channels according to their importance, resulting in improved feature representations. The key advantage of SENet lies in its ability to learn the importance of each channel in an adaptive manner. However, its limitation is the requirement for substantial computational resources, which may hinder its application in certain practical scenarios.

Following the introduction of SENet, several improved channel attention-based models have been proposed, such as the Convolutional Block Attention Module (CBAM) [32], Efficient Channel Attention Network (ECA-Net) [33], and Selective Kernel Networks (SKNet) [34]. CBAM is a plug-and-play module that can be embedded into existing deep learning architectures. It integrates both channel and spatial attention: channel attention adjusts the importance of each channel, while spatial attention refines the importance of each spatial location. This combination enhances model performance and offers greater flexibility compared with other attention-based approaches. ECA-Net is a lightweight model that introduces the ECA module to strengthen the model’s focus on salient features, thereby improving performance while maintaining computational efficiency. SKNet, in contrast, is based on selective convolution. By employing a selective weighting vector to control the receptive field of each convolution kernel, SKNet effectively implements channel attention and improves model performance.

The advantage of channel attention mechanisms lies in their ability to adaptively adjust the importance of each channel and their flexibility to be embedded into existing deep learning models. However, their limitation is the demand for substantial computational resources, which may render them unsuitable for certain tasks, such as real-time processing of large volumes of remote sensing imagery [35], or in scenarios with limited computational capacity, where channel attention-based models may not be the most appropriate option [36]. In addition, when the number of target classes in remote sensing imagery is small or the distinctions between classes are not sufficiently pronounced, the performance of channel attention-based models may also be suboptimal.

The self-attention mechanism has demonstrated remarkable advantages in semantic segmentation tasks in recent years. Unlike conventional CNNs, self-attention overcomes the limitation of local receptive fields by effectively modeling global dependencies between any positions in an image. This capability enables superior performance in capturing complex scene structures and ensuring semantic consistency [37]. In the typical Transformer architecture, input features are processed through multi-head attention modules, which allow the integration of contextual information from spatially distant pixels. This property is particularly beneficial for segmentation tasks involving blurred boundaries or objects with large variations in size [38].

To further enhance the practicality of self-attention in semantic segmentation, numerous studies have proposed innovative architectures that integrate contextual information across different levels. For instance, the Dual Attention Network (DANet) in-corporates both position attention and channel attention, thereby improving the dis-criminative power of feature representations [18]. Another representative method is the Criss-Cross Network (CCNet), which employs a crisscross attention mechanism to achieve near-global modeling while maintaining relatively low computational cost [39]. In addition, the Segmenter model adopts the Vision Transformer (ViT) as its encoder and introduces semantic tokens in the decoder stage to strengthen segmentation performance, further demonstrating the feasibility and superiority of pure Transformer-based architectures for semantic segmentation [40]. Collectively, these studies indicate that self-attention mechanisms, under diverse structural designs, can effectively improve segmentation accuracy and adapt to a wide range of scenarios.

In summary, attention mechanisms play a crucial role in the semantic segmentation of remote sensing imagery. They enable models to capture discriminative features across different regions and channels, thereby improving their accuracy and robustness. With the continuous advancement of deep learning and remote sensing technologies, attention mechanisms are expected to assume an even more significant role in the semantic segmentation of remote sensing images.

Given the inherent limitations of approaches that rely solely on either CNNs or attention mechanisms, in this study, we design a hybrid decoder that integrates self-attention and convolutional networks. Building on this design, we propose the se-mantic segmentation model ACMNet.

## Principles and implementation of ACMNet

### Overview

ACMNet adopts an innovative hybrid architecture consisting of a CNN-based en-coder and an attention-convolution mixed decoder. The decoder is designed with two parallel branches: the self-attention branch, which extracts global contextual information, and the convolution branch, which captures local contextual information. By integrating the outputs of these two branches, the model achieves improved semantic segmentation performance. Within the overall framework, feature maps at different scales are progressively refined, which enables hierarchical aggregation of contextual information, and the final segmentation results are generated. The overall structure of ACMNet is illustrated in Fig 1.

**Fig 1:**
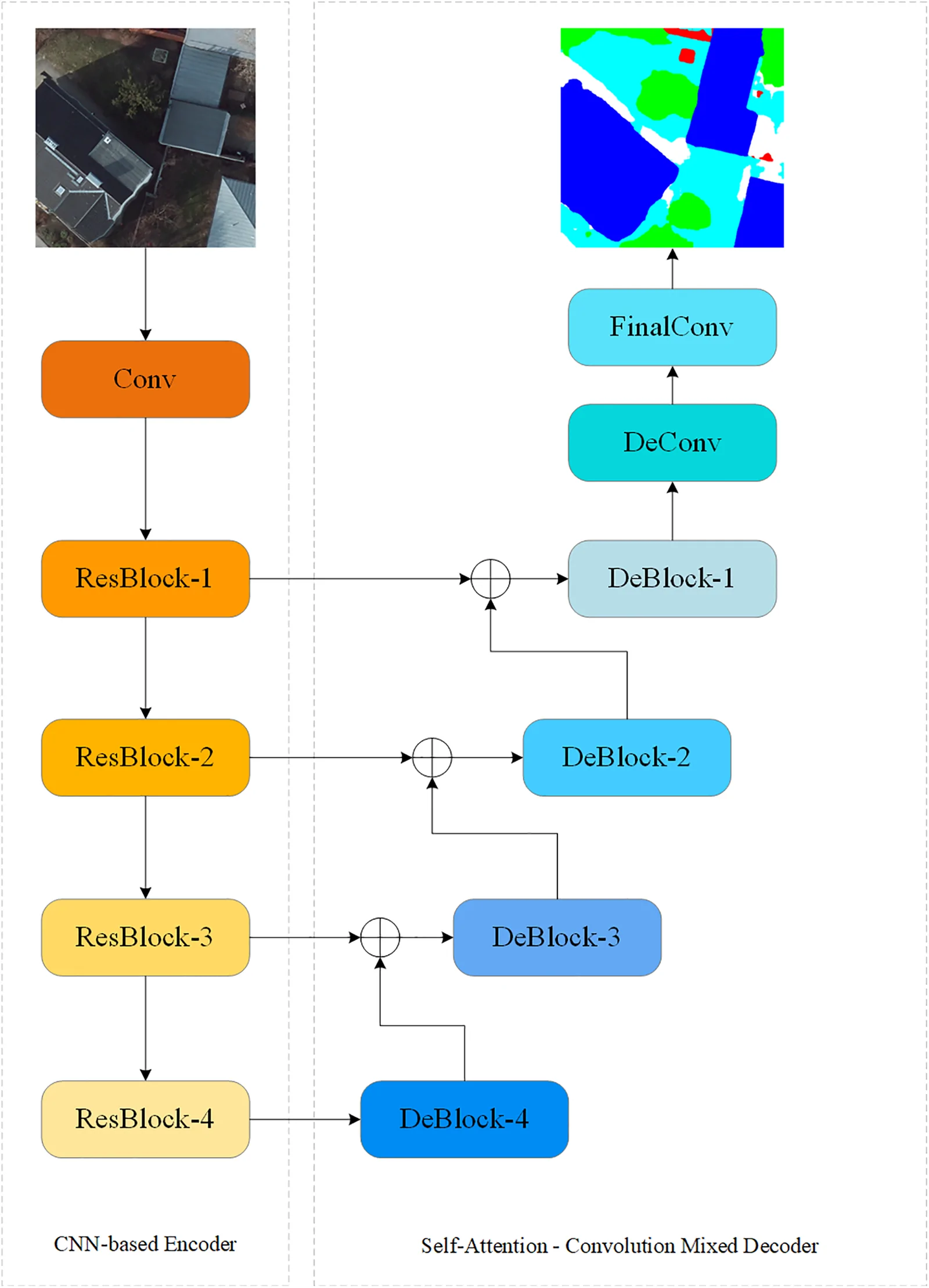
Schematic diagram of the ACMNet architecture.

### CNN-based encoder

Given the effectiveness and efficiency of ResNet-50 in various real-time semantic segmentation tasks, we adopt the pretrained ResNet-50 as the encoder to extract multi-scale semantic features while significantly reducing computational cost.

ACMNet employs a four-stage ResBlock architecture. At each stage, the input feature maps are progressively down-sampled using specific scaling factors, thus enabling the extraction of semantic information at multiple scales. The low-level features obtained are then up-sampled and combined with the feature maps extracted at each stage through weighted summation to achieve information fusion across different feature levels. Subsequently, the fused feature maps are further up-sampled and integrated with high-level semantic features to refine and guide the final feature representation. This process en-hances the model’s ability to restore spatial details and maintain semantic consistency in semantic segmentation tasks.

### Self-attention-convolution mixed decoder

The structural diagram of this module is shown in Fig 2. The self-attention-convolution mixed decoder consists of two parallel branches: a self-attention branch and a convolutional branch. The self-attention branch is mainly used to enhance semantic consistency through global correlation modeling, whereas the convolutional branch is used to preserve local textures, boundary contours, and small-object details. The model then integrates the global contextual information and local contextual information extracted by the two branches to make comprehensive use of these two types of complementary information.

**Fig 2:**
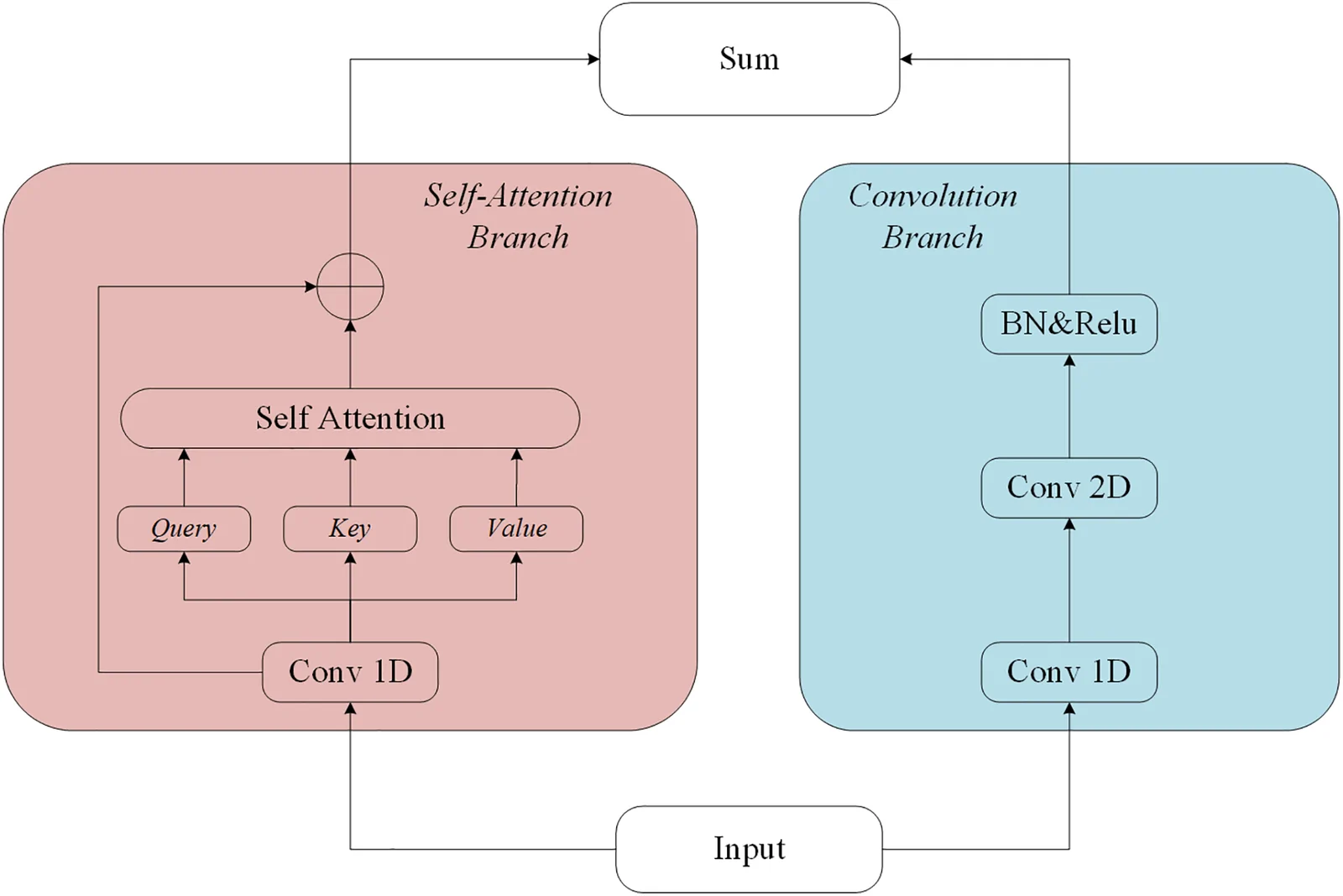
Schematic diagram of the self-attention-convolution mixed decoder.

#### Self-attention branch.

Let *N* and *C* denote the length of the input feature sequence and the number of channels, respectively, where *N* = *H* × *W*, and H and W represent the height and width of the feature map. Given an input feature ($X=\left[x_{\text{1}},x_{\text{2}}\text{\textbackslash{}ldots}x_{N}\right]\in R^{N\times C}$), three projection matrices ($W_{Q}\in R^{D_{X}\times D_{K}}$) are applied to generate the corresponding query (*Q*), key (*K*), and value (*V*) matrices:


$$\begin{array}{c} Q=XW_{q}\in R^{N\times D_{k}} \end{array}$$ (1)

$$\begin{array}{c} K=XW_{k}\in R^{N\times D_{k}} \end{array}$$ (2)

$$\begin{array}{c} V=XW_{v}\in R^{N\times D_{v}} \end{array}$$ (3)

where *Q* denotes the query information used by the current position to actively retrieve relevant features; *K* denotes the key features at each position that can be matched with the query; and *V* denotes the value features to be aggregated. *D*_(·)_ represents the dimension of (·). Since the similarity between *Q* and *K* needs to be calculated in the subsequent step, they should have the same feature dimensionality.

The dot product between *Q* and *K* is then calculated to obtain the similarity matrix *S* =${QK}^{T}$. Each element *S*_*ij*_ in the matrix indicates the degree of correlation between the query feature at the *i*^th^ position and the key feature at the *j*^th^ position. A larger value indicates that the *i*^th^ position needs to refer more strongly to the information from the *j*^th^ position.

To convert the similarity scores into comparable weights, *S* needs to be normalized. A normalization function (ρ) is defined for this purpose. Through $\rho \left(q_{i}^{T}k_{j}\right)\in R^{\text{1}}$, the similarity between the *i*^th^ feature of *Q* and the *j*^th^ feature of *K* can be evaluated. Let the function be defined as:


$$\begin{array}{c} softmax_{row}\left(QK^{T}\right)=\rho \left(QK^{T}\right) \end{array}$$ (4)

Here, *softmax*_*row*_ denotes the application of the *softmax* function to each row of the matrix *QK*^*T*^. By applying the softmax function to the result of the dot product, the attention weights can be obtained. Finally, the resulting attention weights are used to compute a weighted sum of *V*, thereby producing the output features integrated with global contextual information. In other words, the self-attention mechanism does not make predictions based only on a local window around the current position. Instead, it selects more relevant positions from the entire feature map according to the attention weights and aggregates information from them. However, in this computational process, given $Q\in R^{N\times D_{k}}$ and $K^{T}\in R^{D_{k}\times N}$, the similarity matrix $QK^{T}\in R^{N\times N}$. This results in both computational complexity and memory complexity of *O*(*N*^2^). Excessively high computational and memory complexity can reduce the running speed of the model, consume substantial memory resources, and limit model scalability. Therefore, dot-product attention is severely constrained in large-scale semantic segmentation applications.

Intuitively, attention weights can be understood as the degree to which the model refers to other pixels or regions when determining the class of a given pixel or region. For example, when building edges are occluded by shadows, or when low vegetation and trees have similar local textures, the model can assign higher attention weights to distant regions with the same semantics or clearer structural information. This allows the model to improve the stability of class prediction. This mechanism is one of the main advantages of self-attention over purely convolutional operations.

Since the computational complexity of dot-product attention grows quadratically with the input size (*N* = *H* × *W*), and the number of input channels *C* is typically much smaller than the number of pixels in the feature map (*C* ≤ *N*), the complexity of the *softmax* function along the channel dimension is only *O*(*C*^2^), which is relatively low. Based on this observation, this branch is designed to extract global contextual information while simultaneously reducing computational complexity. Similar to the standard dot-product attention mechanism, a residual connection is introduced in this branch, which allows the results obtained through the dot-product operation to be added to the refined features. This operation yields the final output of the corresponding module.

It should be noted that the self-attention branch in this study does not adopt the multi-head attention mechanism commonly used in Transformers. Instead, it models correlations along the channel dimension to reduce the computational cost and GPU memory consumption associated with high-resolution remote sensing image inputs. Therefore, the proposed model does not include the number of attention heads as a tunable hyperparameter.

#### Convolution branch.

Let the input feature map be denoted as $X\in R^{\left(H^{\prime }\times W^{\prime }\times C^{\prime }\right)}$. After applying a standard convolution operator *F*_*tr*_, the transformed feature map ($U\in R^{H\times W\times C}$) is obtained. The operator *F*_*tr*_ is defined as:


$$\begin{array}{c} U_{C}=V_{C}*X=\sum_{S=1}^{C^{\prime }}V_{C}^{S}*X^{S} \end{array}$$ (5)

where *V* represents a set of learnable convolutional filters; *V*_*c*_ denotes the parameters of the *c*^th^ filter; $V_{C}^{S}$ is a two-dimensional convolution kernel; and * indicates the convolution operation.

Once the feature map *U* is generated, a global average pooling function *F*_*sq*_ is applied to compress the feature map into a channel-wise descriptor $Z\in R^{1\times 1\times C}$. The operation is defined as:


$$\begin{array}{c} Z_{C}=F_{sq}\left(U_{C}\right)=\frac{\text{1}}{H\times W}\sum_{i=1}^{H}\sum_{j=1}^{W}U_{C}\left(i,j\right) \end{array}$$ (6)

where Z_*c*_ denotes the *c*^*t*h^ element of *Z*.

Next, a function *F*_*ex*_ is applied to extract channel-wise weight information. Specifically, the channel descriptor vector *Z* obtained in the previous step is processed by two fully connected layers, *W*1 and *W*2, to generate the channel weights *s*. The first fully connected layer uses a ReLU activation, while the second employs a Sigmoid activation. Between the two layers, a reduction ratio *R* is introduced as a hyperparameter. After the first fully connected layer, the dimension of *Z* is reduced from (1 × 1 × *C*) to (1 × 1 × *C*/*R*); after the second fully connected layer, the dimension is restored to (1 × 1 × *C*). The resulting vector *s* contains the weight values for each channel, where different values correspond to the relative importance of different channels:


$$\begin{array}{c} s=F_{ex}\left(Z,W\right)=\sigma \left(g\left(Z,W\right)\right)=\sigma \left(W_{\text{2}}\delta \left(W_{\text{1}}Z\right)\right) \end{array}$$ (7)

Finally, the attention weights *s* are applied to recalibrate the feature maps. Each feature map in *U* is multiplied by its corresponding channel weight, thereby producing the final output $\tilde{X}$ of the squeeze-and-excitation (SE) module:


$$\begin{array}{c} {\tilde{X}}_{C}=F_{scale}\left(U_{C},s_{C}\right)=s_{C}U_{C} \end{array}$$ (8)

In the convolution branch, two cascaded convolutional layers with kernel sizes of 1 and 3 are employed to capture local contextual information. The output is then normalized by batch normalization and activated using ReLU. The resulting features are processed through the SE module to generate the final output of the convolution branch.

#### Algorithm flow.

The algorithmic procedure of ACMNet is summarized in Algorithm 1.

**Algorithm 1.** The algorithm implementation process of ACMNet (ISPRS Potsdam dataset)

Input: I (NIR,R,G,DSM)

Output: P (Prediction results of semantic segmentation)

//Step1: Extracting multi-level feature maps from the encoder

X=Conv(𝐈)

$F_{\text{1}}=ResBlock\text{1}(X)$

$F_{\text{2}}=ResBlock\text{2}(F_{\text{1}})$

$F_{\text{3}}=ResBlock\text{3}(F_{\text{2}})$

$F_{\text{4}}=ResBlock\text{4}(F_{\text{3}})$

//Step2: Perform multi-level fusion of contextual features in decoder

$\text{encoder}\_\text{features }=[F_{\text{1}},F_{\text{2}},F_{\text{3}},F_{\text{4}}][::-\text{1}]$

decoder_outputs =[]

x = encoder_features[0]

for i in range(len(encoder_features)):

 if *i* > 0:

  x=x+ encoder_features[i]

 global_feat = SelfAttentionBranch(x)

 local_feat=ConvolutionBranch(x)

 x=global_feat+local_feat

//Step3: Output prediction results

upsample=DeConv(x)

𝐏=FinalConv(upsample)

## Experimental results and analysis

### Experimental environment

All experiments in this study were conducted under the Windows 10 operating system. The model was implemented in Python 3.10 using the PyTorch 2.1.2 deep learning framework. The experimental platform was equipped with an Intel Xeon Gold 6530 CPU, an NVIDIA TITAN X GPU with 12 GB of memory, and 64 GB of system memory. The number of parameters, FLOPs, GPU memory consumption, inference time per image, and FPS of all models were measured under the same hardware environment. The default input size was 512 × 512, whereas the input-resolution sensitivity experiments were evaluated separately according to their corresponding input sizes to ensure a fair comparison of computational efficiency.

### Dataset

The experiments in this study involved three remote sensing image datasets: ISPRS Potsdam, ISPRS Vaihingen, and WHU Building (Fig 3). The ablation experiments were mainly conducted on the ISPRS Potsdam dataset to analyze the effectiveness of the internal structural design of ACMNet. The comparative experiments were further extended to the Potsdam, Vaihingen, and WHU Building datasets to evaluate the stability of the model across different remote sensing scenarios and task types.

**Fig 3:**
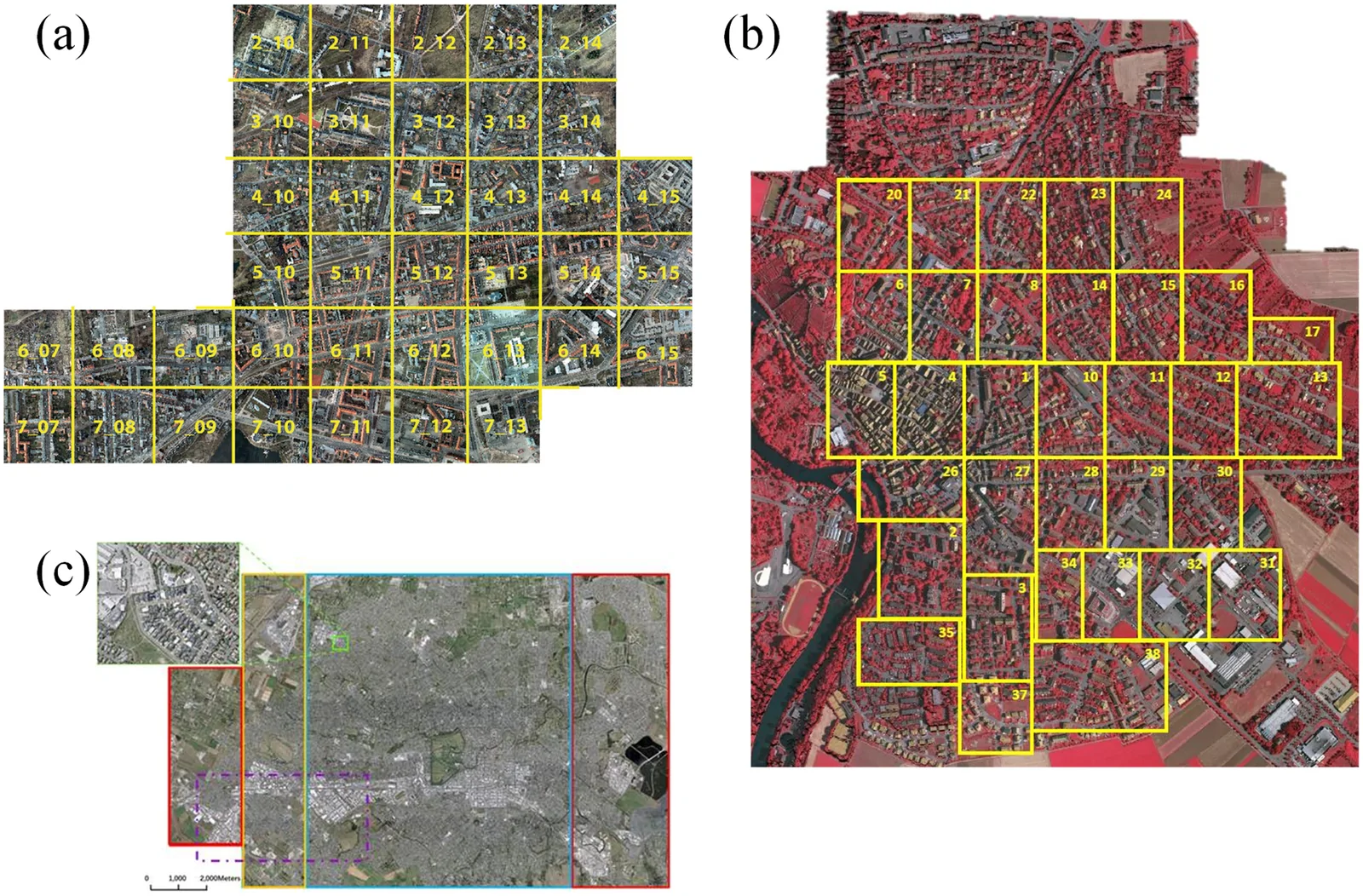
Remote sensing images of the dataset regions. (a) Overview of the Potsdam dataset region. (b) Overview of the Vaihingen dataset region. (c) Overview of the WHU Building dataset region. The numbers in (a) and (b) indicate the identifiers of the images in the corresponding datasets.

The Potsdam dataset provides high-resolution urban remote sensing images acquired by unmanned aerial vehicles. Each image has a spatial resolution of 5 cm and a size of 6000 × 6000 pixels. The dataset contains 38 images, including near-infrared, red, green, blue, DSM, and normalized DSM data. Each image is also provided with corresponding ground-truth labels. The dataset includes six classes: impervious surfaces, buildings, low vegetation, trees, cars, and clutter/background.

In the experiments on the Potsdam dataset, images with the identifiers 2_13, 2_14, 3_13, 3_14, 4_13, 4_14, 4_15, 5_13, 5_14, 5_15, 6_13, 6_14, 6_15, and 7_13 were used for testing. The image with the identifier 20_10 was used for validation, and the remaining 22 images were used for training. Each original image was randomly cropped into 1,000 image patches of 512 × 512 pixels for training.

In the comparative experiments, the ISPRS Vaihingen and WHU Building datasets were also introduced.

The Vaihingen dataset is also designed for semantic segmentation of high-resolution remote sensing images in urban areas and contains 33 remote sensing images. Similar to the Potsdam dataset, these images were acquired by unmanned aerial vehicles and therefore have a high spatial resolution of 9 cm. Each image in this dataset contains NIR, red, green, and DSM data, as well as corresponding ground-truth data. The dataset covers the same semantic classes as the Potsdam dataset. Images with the identifiers 2, 4, 6, 8, 10, 12, 14, 16, 20, 22, 24, 27, 29, 31, 33, 35, and 38 were used for testing. The image with the identifier 30 was used for validation, and the remaining 15 images were used for training.

The WHU Building dataset was manually compiled by the Photogrammetry and Computer Vision Group at Wuhan University. It is designed for tasks such as building extraction and change detection, and includes an aerial imagery building dataset, a global urban satellite imagery building subset, an East Asia satellite imagery building subset, and a building change detection dataset. The aerial imagery part was acquired over Christchurch, New Zealand. The original images have a spatial resolution of 0.075 m and cover an area of approximately 450 km^2^. The official website also provides down-sampled and cropped 512 × 512 image patches together with corresponding raster labels. The aerial imagery samples were cropped into 8,189 image patches and divided into training, validation, and test sets, which contain approximately 130,500, 14,500, and 42,000 building instances, respectively [41].

In the comparative experiments, this study used the WHU Building dataset to evaluate building extraction performance. The labels in this dataset consist of two classes: buildings and background. Unlike the multi-class land-cover segmentation tasks in the Potsdam and Vaihingen datasets, the WHU Building dataset focuses more on the completeness of building regions, the accuracy of boundary contours, and missed or false detections in complex backgrounds. Therefore, in the quantitative comparison on this dataset, this study reports the F1-score and IoU for the background and building classes separately, as well as comprehensive metrics such as OA, mF1, and mIoU, to evaluate the generalization ability of ACMNet in building extraction scenarios.

The three datasets differ in class composition, object scale, and scene complexity. They therefore allow the generalization ability of ACMNet to be evaluated from two perspectives: multi-class land-cover segmentation and binary building extraction.

### Parameter settings and evaluation metrics

#### Training parameter settings.

After multiple rounds of training and testing, the main training parameters were finally determined as follows: the initial learning rate was set to 3e-4, the batch size was set to 4, and the number of epochs was set to 50. The Potsdam and Vaihingen experiments used four-channel inputs composed of RGB/NIR and DSM data, whereas the WHU Building experiments used three-channel RGB inputs. The pixel values of all images were converted to float32 and normalized to the range of [0, 1]. For the Potsdam and Vaihingen datasets, random cropping and random flipping were used for data augmentation during training. The default input crop size was 512 × 512 pixels, and this size was kept unchanged in all experiments except for the input-resolution sensitivity experiment. During validation and testing, sliding-window cropping was used to ensure that the entire image was included in the evaluation. Since the official image patches in the WHU Building dataset are 512 × 512 pixels, random flipping was mainly used for data augmentation during training, while no random augmentation was applied during validation or testing. Model training used the sum of cross-entropy loss and Dice loss as the optimization objective. In the multi-class land-cover segmentation experiments, the Adam optimizer was used, and the learning rate was scheduled with CosineAnnealingWarmRestarts. In the binary building extraction experiments on the WHU Building dataset, the AdamW optimizer was used, with the weight decay set to (5 \times 10^{−4}), and CosineAnnealingLR was used for learning rate decay. During the experiments, the best model was saved according to the validation-set metrics. For the multi-class experiments, the best weights were selected mainly based on the validation mF1. For the WHU Building experiments, the best weights were selected based on the building IoU. To evaluate the sensitivity of the ablation results to random initialization and sample shuffling, the module contribution ablation experiments in Table 1 independently trained FCN, FCN + SAM, FCN + CM, and ACMNet with three different random seeds. The results are reported as the mean ± standard deviation, and the related analysis mainly evaluates model stability based on the mean and standard deviation of the repeated experiments.

**Table 1:** Statistical results of the module contribution ablation experiments under different random seeds on the Potsdam dataset (%).

Method	Number of runs	Impervious surface F1	Building F1	Low vegetation F1	Tree F1	Car F1	mF1	OA	mIoU
FCN	3	89.80 ± 0.48	97.08 ± 0.83	89.06 ± 0.68	80.13 ± 1.23	93.93 ± 0.45	90.00 ± 0.09	87.99 ± 0.40	82.30 ± 0.25
FCN + SAM	3	91.52 ± 0.58	97.28 ± 0.69	88.74 ± 1.07	80.59 ± 0.92	94.08 ± 0.30	90.44 ± 0.22	88.43 ± 1.07	83.04 ± 0.44
FCN + CM	3	91.56 ± 0.60	96.56 ± 1.06	89.34 ± 0.51	80.46 ± 1.19	93.95 ± 0.45	90.37 ± 0.09	88.45 ± 0.18	82.89 ± 0.07
ACMNet	3	90.95 ± 1.53	97.09 ± 0.51	89.77 ± 0.59	80.91 ± 0.93	94.54 ± 0.14	90.65 ± 0.13	88.49 ± 0.05	83.36 ± 0.38

#### Evaluation metrics.

To provide a more objective evaluation of the performance of the proposed ACMNet, this study uses F1-score, overall accuracy (OA), and mean intersection over union (mIoU) as the main evaluation metrics. Among them, F1-score is used to measure the balance between precision and recall for class prediction, OA is used to measure the overall classification accuracy across all pixels, and mIoU is used to measure the average degree of overlap between the predicted regions and the ground-truth regions.


$$\begin{array}{c} \text{F1-score=2}\times \frac{\text{precision}\times \text{recall}}{\text{precision+recall}} \end{array}$$ (9)

$$\begin{array}{c} \text{OA=}\frac{\sum_{k=1}^{N}TP_{k}}{\sum_{k=1}^{N}TP_{k}+FP_{k}+TN_{k}+FN_{k}} \end{array}$$ (10)

$$\begin{array}{c} \text{mIoU=}\frac{\text{1}}{N}\sum_{k=1}^{N}\frac{TP_{k}}{FN_{k}+FP_{k}+TP_{k}} \end{array}$$ (11)

where *TN*, *FN*, *TP*, and *FP* represent the true negatives, false negatives, true positives, and false positives, respectively, with *k* indicating the number of classes.

In addition, this study reports class-level F1-scores to analyze the segmentation performance of the model across different land-cover classes. For the Potsdam and Vaihingen datasets, the tables present the F1-scores for five major land-cover classes: impervious surfaces, buildings, low vegetation, trees, and cars. The corresponding mF1 and mIoU values are also calculated. For the WHU Building dataset, the F1-score and IoU are reported separately for the background and building classes.

### Experimental results and analysis

#### Ablation study.

To more comprehensively evaluate the effectiveness of the proposed self-attention-convolution mixed decoder, this study conducted ablation experiments on the ISPRS Potsdam dataset. An FCN model with ResNet-50 as the encoder was used as the baseline. The self-attention branch in the decoder is denoted as SAM, or Self-Attention Module, and the convolutional branch is denoted as CM, or Convolution Module. On the basis of the module contribution experiment, this study further investigated the fusion weights of SAM and CM, different input resolutions, multi-scale feature fusion levels, and model complexity. These experiments provide a more detailed analysis of the effects of each design factor in ACMNet on segmentation accuracy and computational efficiency.

Table 1 presents the statistical results of the four module configurations under three random seeds. The complete ACMNet achieved mF1, OA, and mIoU values of 90.65 ± 0.13, 88.49 ± 0.05, and 83.36 ± 0.38, respectively, which were the highest values in the table. All three metrics improved compared with FCN, which indicates that the joint design of the self-attention branch and the convolutional branch can stably improve baseline performance under repeated training conditions. After SAM was added alone, mF1 and mIoU increased to 90.44 ± 0.22 and 83.04 ± 0.44, respectively. After CM was added alone, mF1 and mIoU reached 90.37 ± 0.09 and 82.89 ± 0.07, respectively. These results show that both branches can provide effective performance gains. Specifically, SAM is more beneficial for global semantic consistency, whereas CM provides complementary support for local textures and boundary details. Overall, the complete model achieved the best performance in terms of comprehensive metrics. Its standard deviations for mF1 and OA were 0.13 and 0.05, respectively, which suggests that ACMNet has good stability under different random initialization conditions.

To further analyze the interaction between the two branches, this study conducted a sensitivity experiment on the fusion weights of SAM and CM while keeping the other network structures unchanged. As shown in Table 2, balanced fusion (SAM:CM = 1.00:1.00) achieved the highest mF1 and mIoU. When the SAM weight was increased to 0.75, the model performed better than the setting in which the CM weight was increased to 0.75, but it still performed worse than balanced fusion. This indicates that SAM makes a strong contribution to global semantic consistency, while CM supplements local texture and edge information. When the two branches are fused in a relatively balanced manner, the overall performance is more stable.

**Table 2:** Results of the weight sensitivity experiment for the SAM and CM branches.

Weight configuration	mF1	OA	mIoU
SAM:CM = 1.00:1.00	90.61	88.39	83.26
SAM:CM = 0.25:0.75	90.30	87.96	82.78
SAM:CM = 0.75:0.25	90.44	87.85	83.06

The results of the input-resolution sensitivity experiment are shown in Table 3. Compared with the 256 × 256 and 384 × 384 inputs, ACMNet achieved the highest mF1 and mIoU with the 512 × 512 input, reaching 90.61% and 83.26%, respectively. This indicates that a larger input crop can preserve more complete spatial context and object structure information, which helps the model identify details such as building edges, vegetation boundaries, and small vehicles. At the same time, FLOPs and GPU memory consumption increased substantially as the input size increased. The inference time and FPS showed slight fluctuations across different input sizes, which suggests that actual running efficiency can also be affected by hardware scheduling and implementation details. Therefore, in practical applications, a trade-off should be made among accuracy requirements, GPU memory availability, and processing efficiency.

**Table 3:** Performance and computational cost of ACMNet under different input resolutions.

Input size	mF1	OA	mIoU	FLOPs (G)	GPU memory (GB)	Time (ms)	FPS
256 × 256	89.48	88.86	81.31	38.56	0.67	15.69	63.72
384 × 384	89.49	88.30	81.51	86.73	1.73	15.02	66.59
512 × 512	90.61	88.39	83.26	154.18	2.09	15.13	66.08

To verify the role of multi-scale feature fusion, this study compared the results of introducing the mixed module only at the highest semantic level, e4; at e3 + e4; at e2 + e3 + e4; and at all four levels, e1 + e2 + e3 + e4. As shown in Table 4, when only e4 was used, the model achieved an mF1 of 90.17%. After e3 was added, mF1 increased to 90.53%. When e2 was further introduced, the performance showed some fluctuation, which indicates that multi-scale fusion does not improve monotonically simply as the number of fusion levels increases. The complete four-level fusion achieved the highest mF1 and mIoU, reaching 90.61% and 83.26%, respectively. This result shows that high-level semantic features play an important role in overall recognition, while low- and mid-level features can more fully supplement spatial details and boundary information only when they are properly coordinated.

**Table 4:** Results of the ablation experiment on multi-scale feature fusion levels.

Fusion level	mF1	OA	mIoU	FLOPs (G)	Time (ms)	FPS
e4	90.17	87.80	82.60	92.88	11.83	84.55
e3 + e4	90.53	87.76	83.20	113.29	12.10	82.64
e2 + e3 + e4	90.15	87.11	82.58	133.72	13.88	72.05
e1 + e2 + e3 + e4	90.61	88.39	83.26	154.18	15.13	66.08

Table 5 further presents the model complexity and inference efficiency under different module configurations. Compared with FCN, the introduction of either SAM or CM increased the number of parameters and computational cost, with the CM branch producing a more obvious increase in both parameters and FLOPs. With a 512 × 512 input, the complete ACMNet had 135.47M parameters and 154.18G FLOPs, with an average inference time of 15.13 ms per image and an FPS of 66.08. It should be noted that ACMNet did not substantially increase the number of parameters or FLOPs compared with FCN + CM. However, since it retains information interaction between the two branches, its GPU memory consumption and inference time increased to some extent. Overall, compared with the baseline, ACMNet achieved higher comprehensive accuracy at the cost of a certain computational overhead, which reflects a trade-off between accuracy improvement and computational cost.

**Table 5:** Comparison of model complexity under different module configurations with an input size of 512 × 512.

Method	Params (M)	FLOPs (G)	GPU memory (GB)	Time (ms)	FPS
FCN	28.89	72.45	0.89	10.26	97.51
FCN + SAM	79.04	111.16	1.08	12.57	79.55
FCN + CM	135.47	154.16	1.59	13.55	73.79
ACMNet	135.47	154.18	2.09	15.13	66.08

Fig 4 shows the visualization results of the ablation experiments. ACMNet achieved more stable segmentation performance in areas such as low vegetation, trees, and building boundaries. Compared with models that use only a single branch, ACMNet can use both the global contextual information provided by SAM and the local texture and boundary information provided by CM. This enables the model to better distinguish low vegetation from trees when their spectral characteristics are similar and to maintain better spatial integrity along building edges. This result is consistent with the trend in Table 1, where ACMNet achieved the highest mean values for the comprehensive metrics. It further demonstrates that the collaborative design of the two branches can improve the model’s ability to identify complex land-cover boundaries and easily confused classes.

**Fig 4:**
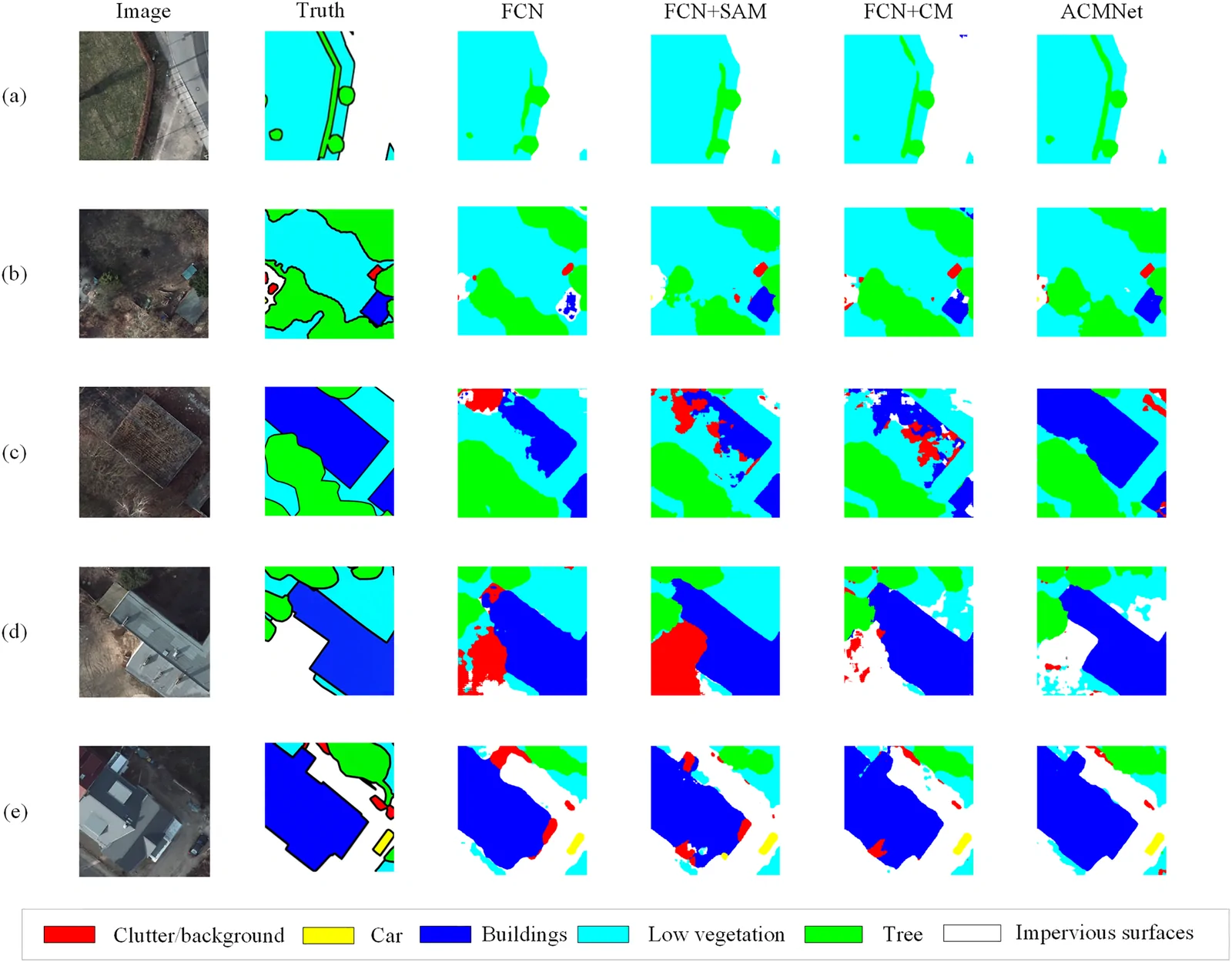
Results of the ablation experiments.

#### Comparative experiments with other models.

To evaluate the performance of ACMNet more fairly, this study quantitatively compared it with several representative semantic segmentation models. The comparison models include FCN, which is based on a fully convolutional structure; DeepLabV3, which uses atrous convolution and spatial pyramid pooling to enlarge the receptive field; DC-Swin, which combines dynamic convolution with the hierarchical feature modeling capability of Swin Transformer; PSPNet, which uses pyramid pooling for global scene parsing; and UNetFormer, which integrates a U-shaped architecture with Transformer modules. These models improve semantic segmentation performance for remote sensing images from different perspectives, including local convolutional modeling, multi-scale context extraction, and Transformer-based global dependency modeling. To avoid conclusions that may arise incidentally from a single dataset, this study conducted comparative experiments on the Potsdam, Vaihingen, and WHU Building datasets. The quantitative results are shown in Tables 6–8.

**Table 6:** Quantitative comparison results of different models on the Potsdam dataset (%).

Method	Impervious surface	Building	Low vegetation	Tree	Car	mF1	OA	mIoU
FCN	92.08	95.95	86.41	87.51	95.78	91.55	89.74	84.66
DeepLabV3	92.74	95.42	86.07	87.61	95.20	91.41	89.13	84.41
DC-Swin	89.22	92.65	83.14	81.02	92.29	87.67	86.16	78.36
PSPNet	92.17	96.02	86.15	87.50	95.79	91.52	89.77	84.63
UNetFormer	92.54	95.73	85.91	87.35	93.26	90.96	89.74	83.63
ACMNet	92.51	96.24	86.55	87.63	96.03	**91.79**	**90.20**	**85.09**

**Table 7:** Quantitative comparison results of different models on the Vaihingen dataset (%).

Method	Impervious surface	Building	Low vegetation	Tree	Car	mF1	OA	mIoU
FCN	90.67	93.99	81.18	88.30	84.54	87.73	88.72	78.43
DeepLabV3	91.44	94.50	81.11	88.01	84.79	87.97	89.04	78.84
DC-Swin	87.66	89.72	78.37	85.96	72.33	82.81	85.44	71.17
PSPNet	90.94	94.42	81.41	88.55	86.96	88.45	89.17	79.57
UNetFormer	91.76	94.44	81.73	88.49	86.44	88.57	89.30	79.77
ACMNet	92.06	94.79	81.39	88.12	87.12	**88.70**	**89.47**	**80.00**

**Table 8:** Quantitative comparison results of different models on the WHU Building dataset (%).

Method	Background F1	Building F1	Background IoU	Building IoU	mF1	OA	mIoU
FCN	99.01	92.18	98.05	85.50	95.60	98.25	91.78
DeepLabV3	98.96	91.82	97.95	84.88	95.39	98.16	91.41
DC-Swin	98.76	89.98	97.54	81.78	94.37	97.79	89.66
PSPNet	99.17	93.28	98.35	87.40	96.22	98.52	92.87
UNetFormer	99.19	93.59	98.40	87.95	96.39	98.57	93.18
ACMNet	**99.23**	**93.89**	**98.48**	**88.48**	**96.56**	**98.64**	**93.48**

Table 6 reports the quantitative comparison results of different models on the Potsdam dataset. ACMNet achieved the best results for all three comprehensive metrics, with mF1, OA, and mIoU values of 91.79%, 90.20%, and 85.09%, respectively. ACMNet also achieved the best performance for the building, low vegetation, and tree classes. Notably, ACMNet reached an F1-score of 96.03% for the car class, outperforming the other comparison models. This indicates that the proposed method enhances the representation of small objects and local geometric details while maintaining its ability to model global context.

The Vaihingen dataset contains more pronounced variations in land-cover scale, with complex spatial interweaving among buildings, roads, trees, and vehicles. As shown in Table 7, ACMNet also achieved the best mF1, OA, and mIoU on this dataset, reaching 88.70%, 89.47%, and 80.00%, respectively. Although PSPNet and UNetFormer showed slight advantages in individual classes such as trees or low vegetation, ACMNet performed more stably in the building and car classes. This suggests that the global semantic information provided by the attention branch and the local spatial details preserved by the convolutional branch can complement each other, thereby improving the overall segmentation reliability of the model in complex urban areas.

Table 8 shows the building extraction results of different models on the WHU Building dataset. This dataset mainly consists of two classes, buildings and background. Therefore, building F1, building IoU, and overall mIoU can more directly reflect the ability of each model to extract building boundaries and main building regions. The experimental results show that ACMNet achieved OA, mF1, and mIoU values of 98.64%, 96.56%, and 93.48%, respectively, while its building F1 and building IoU reached 93.89% and 88.48%, respectively. These values outperformed those of the other comparison models. This indicates that, in scenes where building boundaries are relatively regular but roof textures, shadows, and background interference are complex, ACMNet can reduce boundary fragmentation and local omission while maintaining the continuity of main building regions.

To compare the segmentation effects of different models more intuitively, this study further visualized the prediction results of different models on the three datasets. The areas with the most obvious differences among models are marked in pink, as shown in Figs 5–7. As shown in Fig 5, on the Potsdam dataset, ACMNet better preserves the accuracy of building boundaries and reduces class confusion in local regions. As shown in Fig 6, on the Vaihingen dataset, vehicles and roads are relatively small in scale and densely distributed. ACMNet not only better preserves building boundaries but also provides more complete recognition of small objects such as vehicles. As shown in Fig 7, on the WHU Building dataset, the prediction results of ACMNet for building edges and main building regions are closer to the ground-truth labels. Overall, the quantitative and visualization results show that ACMNet achieves a good balance between global contextual information and local geometric details and therefore demonstrates stronger segmentation ability in key areas such as easily confused classes, small objects, and building boundaries.

**Fig 5:**
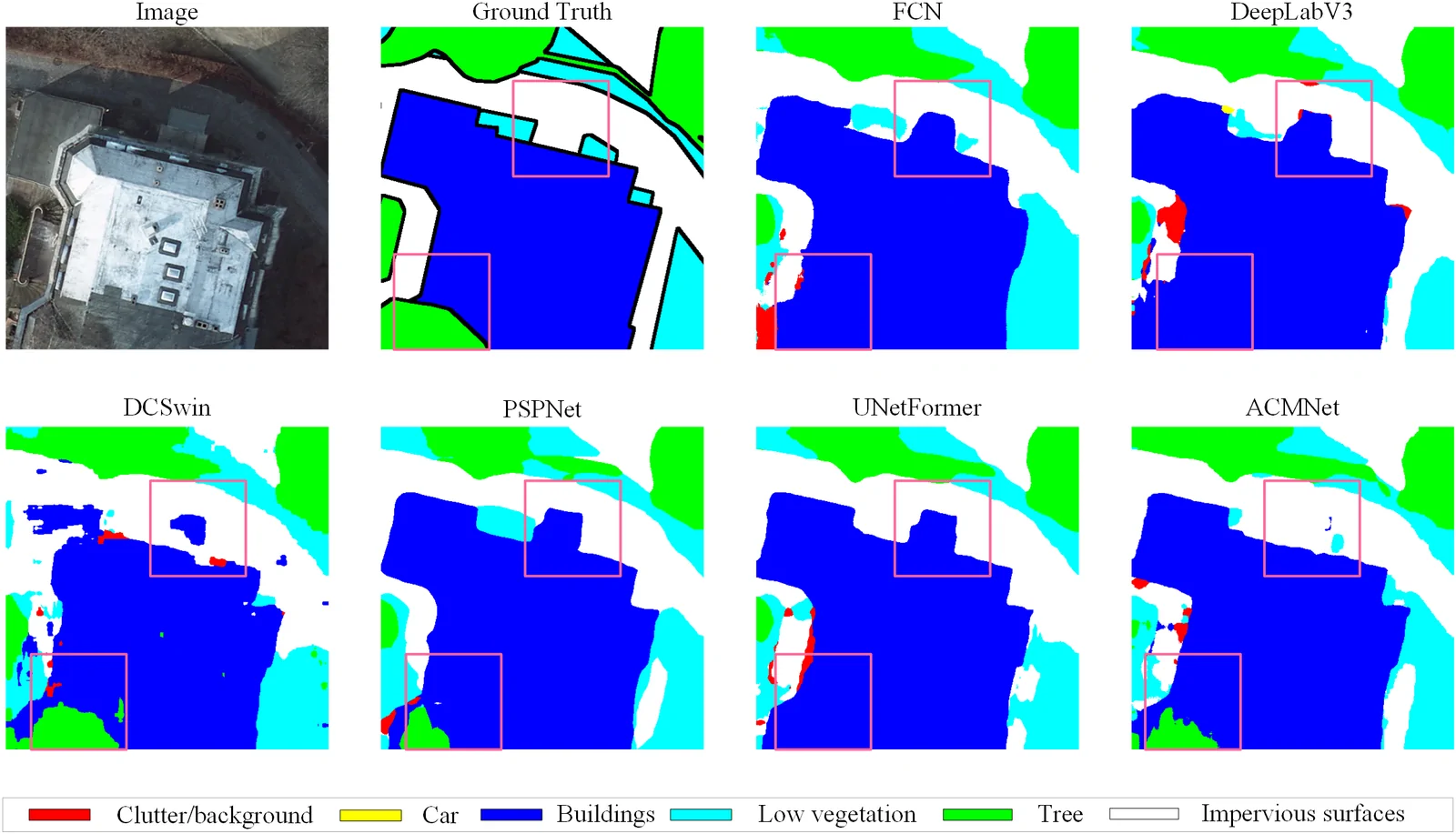
Visualization results of model comparisons on the Potsdam dataset. The pink regions indicate the areas with the most pronounced differences among the prediction results of different models.

**Fig 6:**
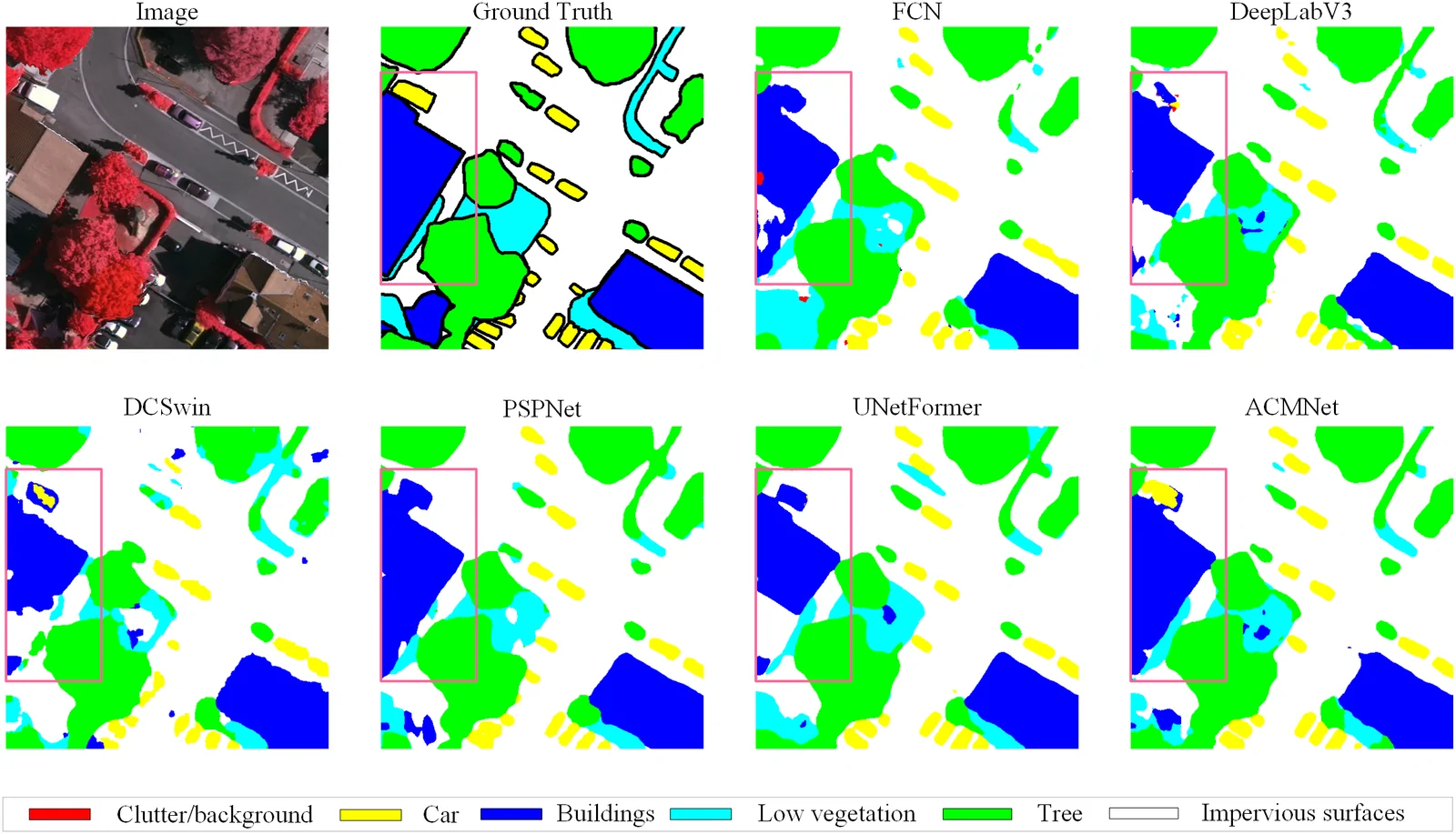
Visualization results of model comparisons on the Vaihingen dataset. The pink regions indicate the areas with the most pronounced differences among the prediction results of different models.

**Fig 7:**
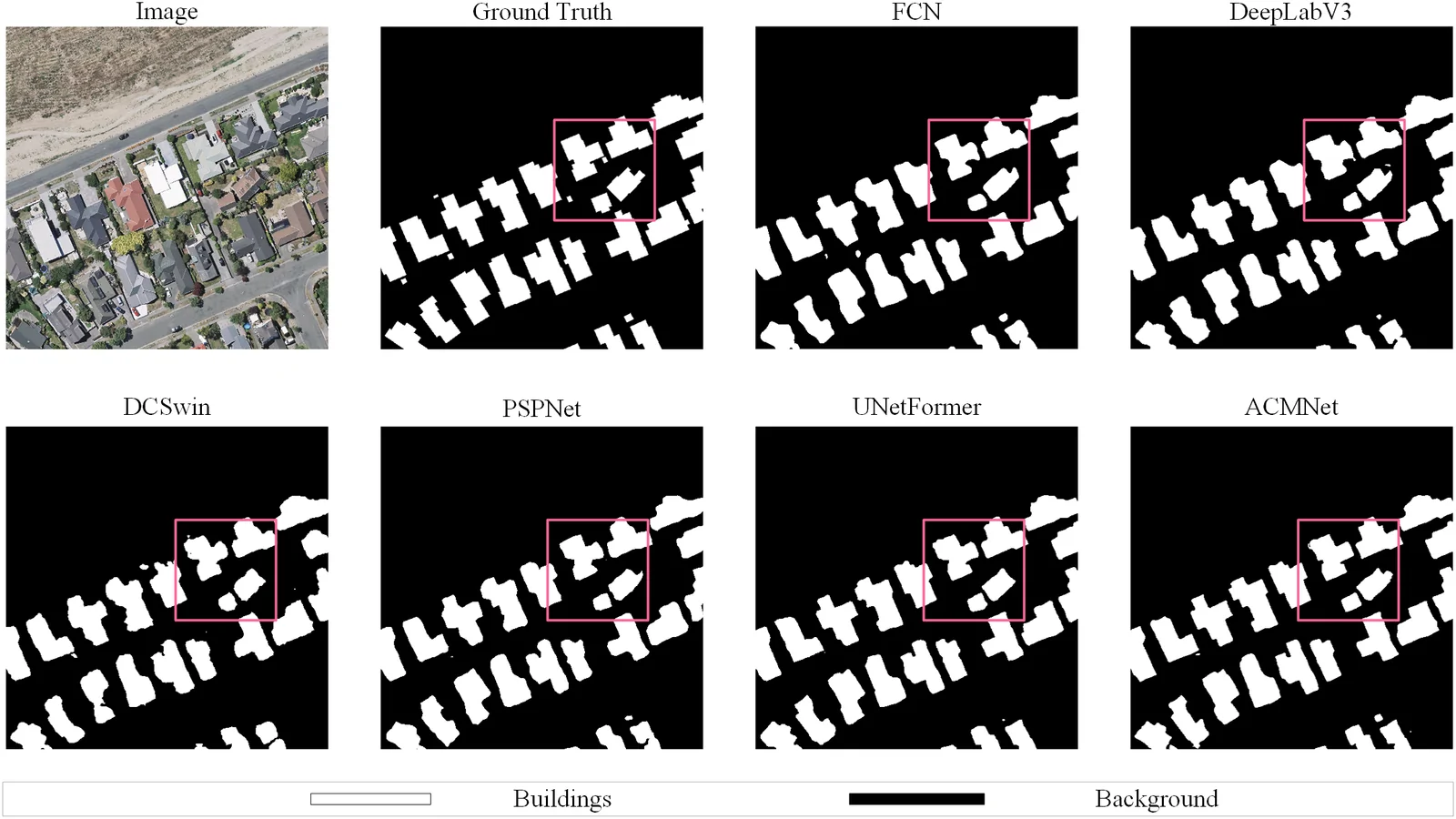
Visualization results of model comparisons on the WHU Building dataset. The pink regions indicate the areas with the most pronounced differences among the prediction results of different models.

#### Summary of experimental results.

Overall, the ablation experiments show that the self-attention branch, convolutional branch, and multi-scale feature fusion jointly contribute to the improved segmentation performance of ACMNet. Under three random-seed runs, the complete ACMNet still achieved the highest average mF1, OA, and mIoU, which indicates that the proposed structure has good stability under repeated training conditions. At the same time, input resolution and module complexity also affect the balance between accuracy and efficiency. The comparative experiments further demonstrate that ACMNet achieved the best comprehensive evaluation results on all three datasets: Potsdam, Vaihingen, and WHU Building. Specifically, ACMNet achieved mF1 values of 91.79%, 88.70%, and 96.56%, OA values of 90.20%, 89.47%, and 98.64%, and mIoU values of 85.09%, 80.00%, and 93.48% on the three datasets, respectively. These results show that, by integrating global semantic context with local spatial details, ACMNet can not only improve the overall segmentation accuracy of high-resolution urban remote sensing images but also enhance model stability across different datasets and object morphologies.

## Discussion

### Role and challenges of introducing self-attention

The experiments demonstrate that incorporating a self-attention mechanism into the decoder effectively captures global contextual information, thereby enhancing the model’s ability to understand global semantics in images and improving segmentation performance. This partially mitigates the limitations of CNNs, which primarily rely on local regions for image understanding and consequently hinder model performance. However, if biased or erroneous low-level features are already extracted in the initial stages due to the restricted local receptive field, it remains uncertain whether the introduction of global context modeling in later stages can sufficiently correct these errors.

This issue originates from the high sensitivity of semantic segmentation tasks to pixel-level semantic consistency and the preservation of spatial structures. Once biased representations are generated in the early stages, particularly under challenging conditions such as blurred object boundaries, subtle inter-class differences, or occlusion, the subsequent modules, even with global modeling capabilities, may fail to accurately recover semantic structures and may even amplify errors. Moreover, although self-attention mechanisms exhibit strong performance in capturing long-range dependencies and global contextual information, their substantial computational cost and large parameter size impose practical constraints on real-world applications.

Therefore, the design of semantic segmentation models should not overly rely on global context enhancement modules in the later stages but should instead place greater emphasis on the accuracy and integrity of feature representations during the feature extraction phase. Specifically, this can be achieved by expanding the receptive field of CNNs, integrating multi-scale feature information, or introducing lightweight attention-guided mechanisms. These strategies can improve feature quality at the source and provide a more robust semantic foundation for subsequent context modeling. Only in this way can models achieve more robust and fine-grained segmentation performance in complex environments.

From the perspective of practical application, the computational overhead introduced by self-attention can directly affect deployment efficiency in tasks such as fine-grained urban land-cover mapping, emergency disaster assessment, building change detection, and dynamic monitoring of land resources. These tasks usually require batch processing of large-scale high-resolution images, and some scenarios also require near-real-time updates. If a model has high GPU memory consumption or slow inference speed, it can increase engineering deployment costs and limit its use on edge devices or resource-constrained platforms. To address this issue, future work can improve the model in three aspects. First, lightweight attention strategies, such as window-based attention, sparse attention, or low-rank approximation, can be adopted to reduce the cost of global modeling. Second, block-wise inference and multi-scale sliding-window strategies can be combined to control the input size of each inference pass while maintaining boundary continuity. Third, model pruning, knowledge distillation, or lightweight backbone networks can be used to further reduce the number of parameters, thereby improving the usability of ACMNet in practical remote sensing processing workflows.

### Limitations

This study proposed ACMNet, a semantic segmentation model that integrates attention mechanisms with convolutional neural networks. By effectively combining global and local contextual information, ACMNet improves the performance of semantic segmentation for remote sensing images. However, the current model still has several limitations. First, although this study validated ACMNet on the Potsdam, Vaihingen, and WHU Building datasets, these datasets are mainly oriented toward high-resolution urban scenes or building extraction tasks. Its adaptability to rural areas, regions dominated by natural land-cover types, and cross-city or cross-sensor conditions still requires further investigation. Second, Potsdam and Vaihingen contain auxiliary information such as NIR and DSM data, whereas WHU Building mainly uses RGB images. This study has not systematically analyzed the effects of different spectral combinations, multispectral inputs, or hyperspectral inputs on model performance. Therefore, the stability of ACMNet under more diverse band configurations remains to be verified. Third, ACMNet enhances global context modeling through the self-attention branch, but local omissions, boundary discontinuities, or confusion between adjacent classes may still occur in highly heterogeneous urban areas, areas with severe shadow occlusion, regions with fragmented building boundaries, and scenes with dense distributions of small objects such as vehicles. Finally, the introduction of the self-attention and convolutional branches increases the number of parameters, GPU memory consumption, and inference time. Although this study has reported model complexity and inference efficiency, further lightweight design is still needed for rapid processing of large-scale remote sensing images or deployment on resource-constrained devices. Future work will further introduce rural, suburban, and cross-regional remote sensing datasets; validate model generalization under multispectral and hyperspectral input conditions; and combine lightweight attention mechanisms, model pruning, or emerging Mamba architectures to optimize the computational efficiency and deployment applicability of ACMNet.

## Conclusions

This study focused on semantic segmentation of high-resolution remote sensing imagery based on a hybrid framework of attention mechanisms and CNNs. We first systematically reviewed the recent research background and development of semantic segmentation in remote sensing and highlighted the limitations of prior models that relied solely on either attention mechanisms or CNNs. We further emphasized the im-portance of effectively integrating the two approaches for enhanced segmentation performance. To address this, we proposed ACMNet, an innovative hybrid model comprising a CNN-based encoder and a self-attention-convolution mixed decoder. The overall structure, underlying principles, and operational mechanisms of ACMNet were described in detail. Finally, ablation experiments were conducted on the ISPRS Potsdam dataset, and comparative experiments were further conducted on the Potsdam, Vaihingen, and WHU Building datasets. These experiments provided a systematic evaluation of the structural effectiveness, generalization ability, and model characteristics of ACMNet.

The results show that ACMNet achieved strong performance in both the ablation experiments with the FCN model and the comparative experiments with FCN, DeepLabV3, DC-Swin, PSPNet, and UNetFormer. Specifically, ACMNet achieved mF1 values of 91.79%, 88.70%, and 96.56% on the Potsdam, Vaihingen, and WHU Building datasets, respectively. Its OA values were 90.20%, 89.47%, and 98.64%, and its mIoU values were 85.09%, 80.00%, and 93.48%, respectively. The experimental results indicate that, for trees and low vegetation with similar spectral characteristics, ACMNet can capture subtle semantic differences between them by integrating global contextual information. This enables the model to accurately distinguish low vegetation from land-cover objects with similar spectral reflectance. ACMNet also provides more accurate building recognition and better preserves the integrity of building edges. In addition, ACMNet maintains strong recognition performance across different classes in more complex scenes.

When it comes to application prospects, ACMNet can provide technical support for various practical remote sensing tasks. For example, in refined urban management, the model can be used for high-precision extraction of land-cover objects such as buildings, roads, vegetation, and vehicles, thereby providing spatial information for urban expansion monitoring, identification of illegal construction, and infrastructure renewal. In building extraction and change detection tasks, the model’s ability to preserve the completeness of main building regions and the continuity of building boundaries can help improve the quality of building footprint mapping. In disaster assessment and emergency response, more accurate land-cover segmentation results can support the identification of key information, such as damaged buildings, blocked roads, and changes in vegetation cover. With further improvements in lightweight design and cross-regional generalization, ACMNet is expected to be applicable to large-scale land-cover mapping, dynamic urban monitoring, and intelligent interpretation of high-resolution remote sensing imagery.
